# Visual stimulation-induced mild stress enhances cognitive behavior in cynomolgus monkey

**DOI:** 10.1038/s41598-018-22136-9

**Published:** 2018-02-28

**Authors:** Dong Ho Woo, Eun Ha Koh, Seung-Hyuk Shin, Young-Su Yang, Jae Chun Choe, C. Justin Lee, Su-Cheol Han

**Affiliations:** 1 Animal Model Research Center, Korea Institute of Toxicology, KRICT, Jeongeup, Republic of Korea; 2 General Toxicology Research Center, Korea Institute of Toxicology, KRICT, Jeongeup, Republic of Korea; 3 Analytical Research Center, Jeonbuk Department of Inhalation Research, Korea Institute of Toxicology, KRICT, Jeongeup, Republic of Korea; 4 Team of Research Planning and Management, Bureau of Ecological Research, National Institute of Ecology, Seocheon, Republic of Korea; 5 Center for Glia-Neuron Interaction, Korea Institute of Science and Technology, Seoul, Republic of Korea; 6 Laboratory of Behavior and Ecology, Division of EcoScience, Ewha Womans University, Seoul, Republic of Korea

## Abstract

Cortisol is a well-known endogenous glucocorticoid that serves as a stress indicator. It is normally released under stressful condition to warn about imminent danger and thus is critical for survival of the species. However, it is unclear how cortisol relates to cognitive process under physiological condition in high-order primates such as non-human primates (NHP). Here, we report that a slight but significant increase in blood cortisol level by mild stress is positively correlated with the cognitive function in cynomolgus monkey. We stimulated 3 groups of monkeys by viewing consecutive series of pictures of monkeys, pictures of humans, or animation still pictures. We first found that the blood cortisol level was significantly higher during the stimulation session and returned to normal after stimulation session. Among the three types of pictures, the monkeys which were stimulated with monkey pictures showed the most significant increase in cortisol level during stimulation. Furthermore, the monkeys showed significantly enhanced manipulation, suggesting that cortisol affected cognitive processes. Overall, our study demonstrates that visual stimulation both increases blood cortisol and enhances manipulating behavior. Therefore, unlike the common notion that cortisol is a stress indicator, our data supports that a mild increase of cortisol enhances cognition in NHP.

## Introduction

In laboratory settings Cynomolgus monkey (C. monkey, *Macaca fascularis*) is mainly used for the final stage of non-clinical research as a confirmation prior to clinical research, because they share many similarities to human in physiology and genetic background^1^. Like humans, monkeys are social animals requiring social interaction. However, most of laboratory monkeys are maintained in individual cage. Yet, there has been very few published information on animal welfare of individually housed C. monkeys.

Visual enrichment is a partial substitute for social interaction and welfare of laboratory monkeys. Visual stimulation increases locomotion and reduces sleeping behaviors of captive rhesus monkeys^2^. The contents of visual stimulation includes various stimulations such as consecutive still pictures and movies. Still pictures^3,4^ and movies^5,6^ have been shown to change the behaviors of non-human primates. However, the detailed information on types of visual stimulation is lacking. A previous study by Ogura and Matsuzawa demonstrated that time of touching pad for indicating individual’s interest is different on viewing them with contents of varied visual stimulations such as human, animation, and different type of monkey pictures, suggesting that each individual may have a visual preference^7^. Therefore, it would be more desirable that the contents of video for improving visual enrichment for individually-housed monkeys be prepared by pre-identification of visual preference.

Cortisol, endogenous glucocorticoid, affects the brain throughout the whole lifespan of an animal from prenatal to adult period. Under stress and certain cognitive processes such as anxiety^8^ and depression^9^ the hypothalamus-pituitary-adrenal (HPA) axis is activated to produce and release glucocorticoid from adrenal glands^10^. It has been demonstrated that rhesus monkey needs 28 days to recover from repeated chair training stress and during this time the cortisol level ranged from 50 to 40 μg/dl on average^11^. It is well known that stress during transportation of a monkey causes to increase blood cortisol level^12^. Serum cortisol concentration is known to oscillate between 150 and 600 ng/ml, and all monkeys tend to show rhythmicity in the blood concentration of cortisol. On the other hand, glucocorticoid injection in lateral amygdala, a temporal lobe functioning emotional arousal of memory consolidation, blocks post-training systemic-dexamethasone-induced memory-enhancement, whereas injection into central nucleus does not block, suggesting that glucocorticoid receptors in lateral amygdala contribute to memory consolidation^13^. Furthermore, mild electrical foot-shock decreases escape latency time and increases time spent in target quadrant, suggesting that small increase of cortisol relates to cognitive processes in Swiss albino mouse^14^. These previous findings raise a possibility that a visual stimulation induces changes in blood cortisol level and cognition-based behaviors.

The aim of the study is to test whether mild stress is involved in various behaviors, such as foraging and manipulation. The foraging behavior is defined as using hand or mouth to put food in mouth, whereas the manipulation behavior is defined as using movement of digit, hand grasps to play with steel-ball and super rubber tube, excluding the movement of hands to mouth^15^. Because digits and hand movements preclude complex and diverse brain activities such as planning, prediction, and execution of motor behaviors, the behaviors using digits reflect mental status and higher order behaviors such as cognition^16^.

## Results

### Visual stimulations increase blood cortisol level

**Figure 1:**
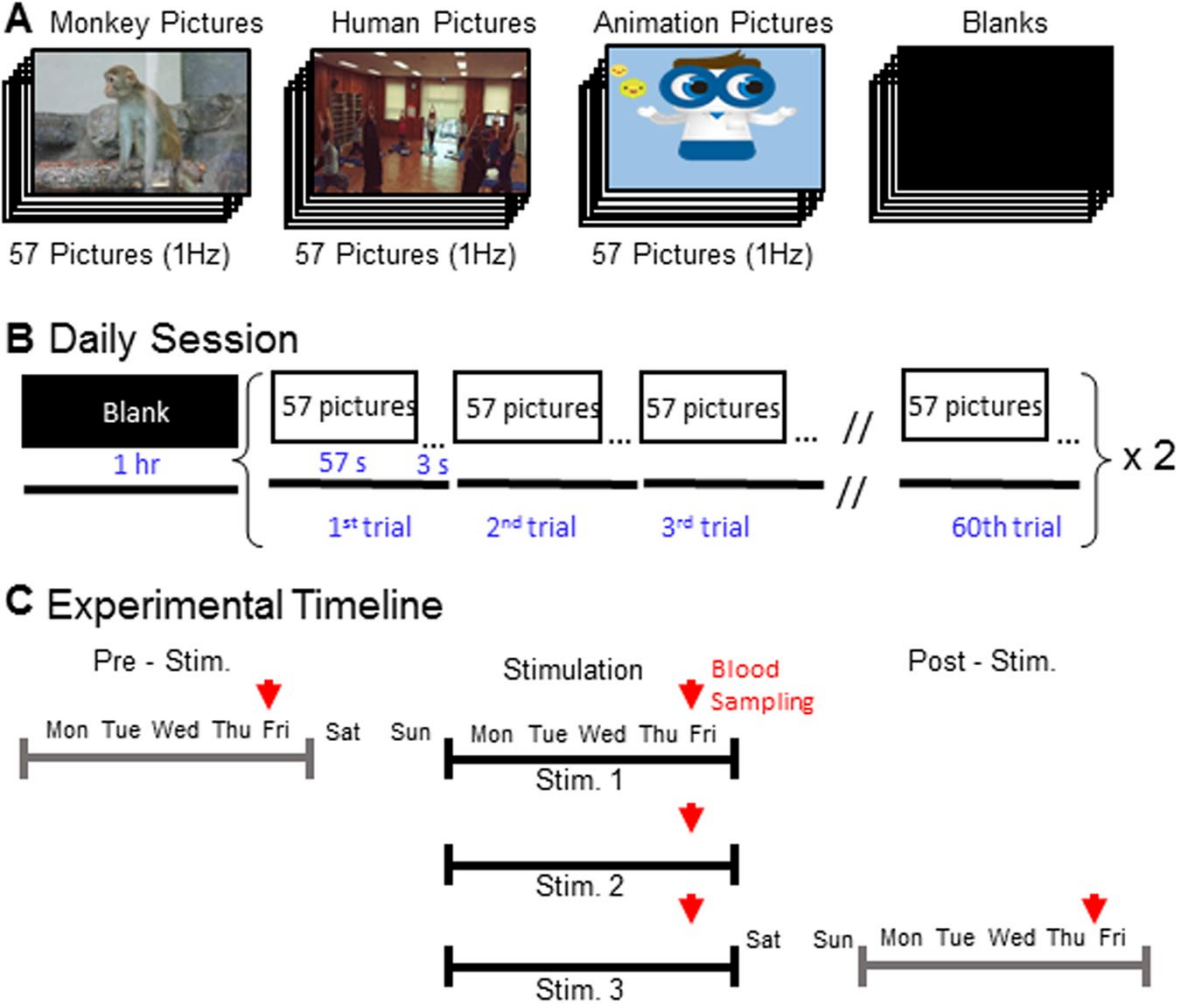
Method of visual stimulation and experimental procedure. (**A**) Contents of visual stimulation, monkey still picture, human still picture, animation still picture, and blank picture. 57 pictures are prepared for 1 trial. (**B**) Daily session is for 3hrs per day. Blank images for 1hr, and visual stimulations such as Monkey, human, and animation pictures are for 1hr 2 times. Each trial is for 1 min, 57 seconds for 57 pictures and 3 seconds for blank. The session of visual stimulation composes of 60 trials. (**C**) Experimental timeline. 4 days of 1 week for 1 pre-stimulation, 4 days of 1 weeks 3 times for visual stimulations, and 4 days of 1 week for 1 post- stimulation. Red arrows indicate collecting time of blood for analyzing cortisol.

All 12 monkeys in this study were age-matched male (Table 1). They were habilitated by the institutional guidelines of KIT. The contents of visual stimulation were categorized into three categories which include consecutive pictures for monkey, consecutive still pictures for human, and consecutive animation pictures for human, and control blank images (Fig. 1A). Subjects were visually stimulated during a daily session. Each session was composed of one-hour stimulation with blanks followed by two-hour stimulation with pictures, each hour of 60 trials. Each trial composed of 57 pictures for 57 s and blanks for 3 s. During a daily session, each monkey was video-recorded and behaviors were analyzed off-line (Fig. 1B). During a week of pre-stimulation and a week of post-stimulation periods, monkeys were shown only with blanks. After pre-stimulation week, actual visual stimulation continued for 3 weeks. (Fig. 1C). Blood samples were collected from monkeys at 5 time points (Fig. 1C), one at the end of pre-stimulation week, three at each end of stimulation weeks, and one at the end of post-stimulation week (Fig. 1C).

**Table 1:** The information of individual C. monkey.

Individuals	Group	Sex	Age(Years)	Body weight(kg)
1	Monkey Pictures	Male	3~5	3~4
2	Monkey Pictures	Male	3~5	3~4
3	Monkey Pictures	Male	3~5	3~4
4	Monkey Pictures	Male	3~5	3~4
5	Human Pictures	Male	3~5	3~4
6	Human Pictures	Male	3~5	3~4
7	Human Pictures	Male	3~5	3~4
8	Human Pictures	Male	3~5	3~4
9	Animation Pictures	Male	3~5	3~4
10	Animation Pictures	Male	3~5	3~4
11	Animation Pictures	Male	3~5	3~4
12	Animation Pictures	Male	3~5	3~4

**Figure 2:**
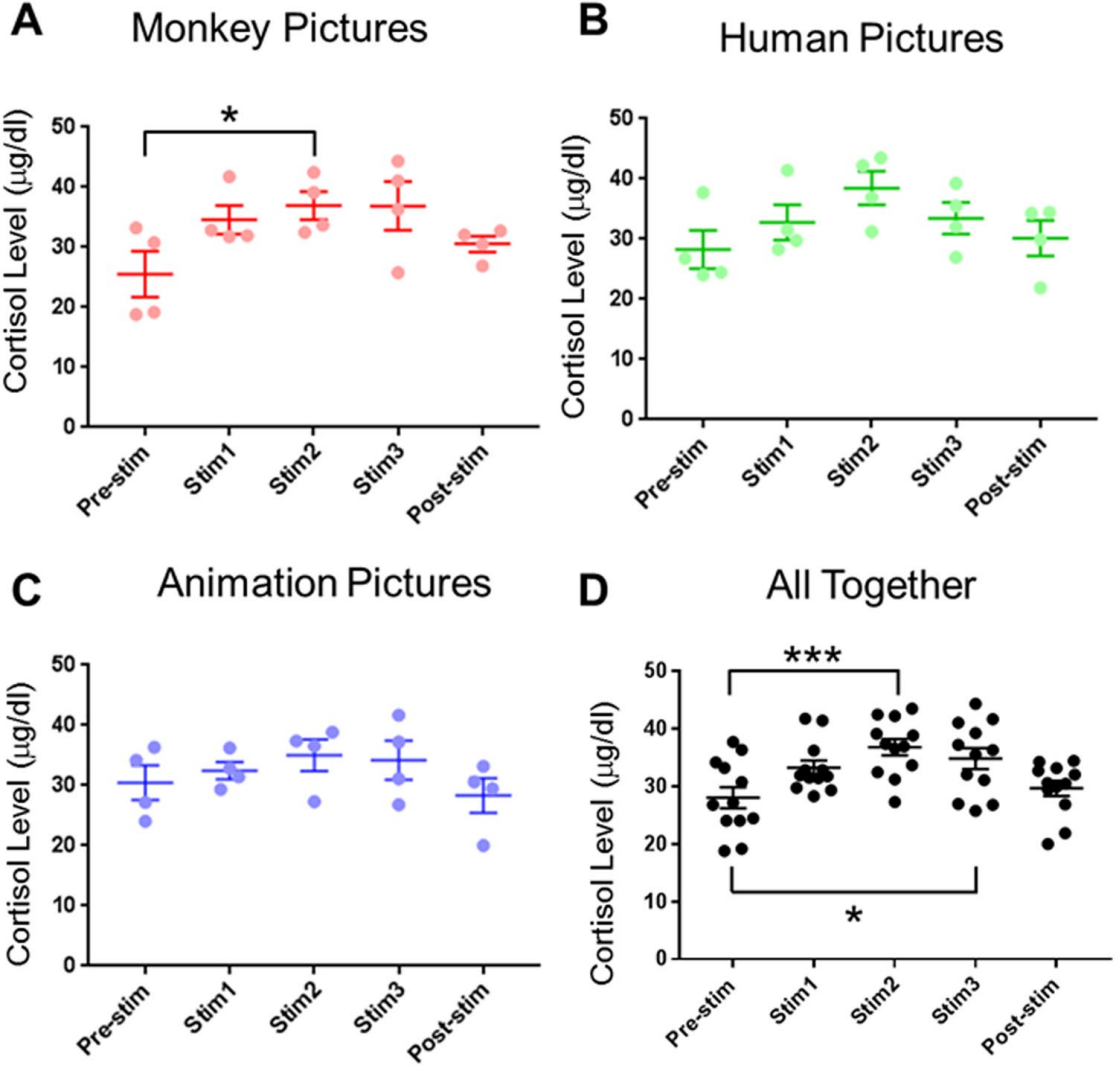
Blood cortisol level of cynomolgus monkey with visual stimulation of (**A**) monkey pictures, (**B**) human pictures, and (**C**) animation pictures. (**A**) Cortisol level is significant in the presence of 2^nd^ stimulation with monkey pictures (one-way ANOVA, F_4,19_ = 2.72, p = 0.07, followed by Dunnett’s posthoc test, *p < 0.05). (**D**) All data are summed (one-way ANOVA, F_4,59_ = 5.43, p = 0.0009, followed by Dunnett’s posthoc test, *p < 0.05, ***p < 0.001). Blood cortisol concentrations with 2^nd^ and 3^rd^ visual stimulation are significantly increased.

A previous study reported that consecutive pictures combined with human, animation, and monkey pictures tended to reduce abnormal behaviors, suggesting that visual stimulation is one of the substitutes for social requirements and beneficial for the welfare of single housed monkey^7^. Because visual enrichment can change behaviors of monkeys, we asked whether hormonal changes such as changes in cortisol level mediate the behavioral changes. To assess blood cortisol concentration, we collected blood samples to see whether cortisol dynamics is changed or not. We found that in general the blood cortisol level increased during simulation weeks compared to pre-stimulation week and returned to the level of pre-stimulation week during post-stimulation week, regardless of types of pictures used for stimulation (Fig. 2). Interestingly, cortisol level in monkeys with viewing monkey pictures was significantly increased at 2^nd^ stimulation week compared to that of pre-stimulation week (One-way ANOVA, F_4,19_ = 2.72, P = 0.069, followed by Dunnett’s posthoc test, *p < 0.05). However, cortisol level from the subjects with human (Fig. 2B) and animation pictures (Fig. 2C) did not show any statistical significance, although the same trend was present. This lack of statistical significance could be due to small number of samples. Thus we added all values (Fig. 2D) and found that cortisol level during 2^nd^ and 3^rd^ stimulation week was significantly increased (One-way ANOVA, F_4,19_ = 5.43, P = 0.0009, followed by Dunnett’s posthoc test, ***p < 0.001, *p < 0.05), indicating that visual enrichment increased blood cortisol level.

### The observed cortisol level suggests visual enrichment causes a mild stress

The previously reported concentration of blood cortisol level ranges from 150 ng/ml to 600 ng/ml in male C. monkeys^17^. The blood cortisol level due to transportation stress in C. monkeys was shown to increase to 700 ng/ml, which recovered to 300 ng/ml^12^. The repeated chair training was shown to increase the cortisol level to 500 ng/ml, which recovered to 400 ng/ml in rhesus monkey^11^. The observed cortisol level ranged from 281 ± 64 ng/ml (pre-stimulation) to 368 ± 49 ng/ml (2^nd^ stimulation) and 348 ± 63 ng/ml (3^rd^ stimulation) (Fig. 2D). Observed increase during visual stimulation was only about 24% of the pre-stimulation level (Fig. 2D). This is consistent with the diurnal oscillation in basal cortisol level in single-housed C. monkey and with the ratio of morning/night cortisol level, which is about 1.3^18^. Therefore, the mild increase in cortisol level during visual stimulation represents a state of mild stress during normal physiological condition in C. monkey. Consistent with this idea, the biochemical analysis of the serum did not show any significant change in other endogenous chemicals and known biomarkers (Table 2)^19^.

**Table 2:** Basic chemical analysis from blood of C. monkey during whole experiment.

Test Item	Groups	Pre stim.	1^st^ stim.	2^st^ stim.	3^st^ stim.	Post-stim.
GLU mg/dL	Monkey	69.6 ± 11.9	67 ± 16.1	69.6 ± 8.9	76.6 ± 16.9	66.3 ± 8.2
	Human	58.9 ± 13.2	66.7 ± 3.5	65 ± 6.8	64.4 ± 7.6	69.3 ± 11.7
	Animation	68.9 ± 12.1	65.8 ± 11.4	62.7 ± 14.4	61.7 ± 15.5	52.8 ± 21.6
BUN mg/d	Monkey	21.5 ± 2.2	21.9 ± 2.8	21.8 ± 2.2	22.1 ± 2.2	19.8 ± 1.9
	Human	25.3 ± 8.05	24.3 ± 4.6	23.8 ± 6.5	25.02 ± 2.3	19.9 ± 3.8
	Animation	24.9 ± 1.8	23.9 ± 1.7	24.8 ± 2.4	25.1 ± 2.1	24.3 ± 4.6
CREA	Monkey	0.9 ± 0.08	0.8 ± 0.06	0.9 ± 0.07	0.9 ± 0.1	0.9 ± 0.1
	Human	0.9 ± 0.1	0.8 ± 0.2	0.8 ± 0.2	0.9 ± 0.1	0.9 ± 0.2
	Animation	0.8 ± 0.08	0.9 ± 0.09	0.8 ± 0.1	0.8 ± 0.06	0.9 ± 0.1
TP	Monkey	7.7 ± 0.3	8 ± 0.5	7.9 ± 0.2	7.7 ± 0.2	7.6 ± 0.2
	Human	7.6 ± 1.1	7.7 ± 0.6	8.1 ± 0.4	8.1 ± 0.6	8.4 ± 0.5
	Animation	7.8 ± 0.4	7.9 ± 0.3	7.7 ± 0.2	7.9 ± 0.6	7.9 ± 0.5
ALB	Monkey	4.2 ± 0.08	4.3 ± 0.2	4.3 ± 0.1	4.2 ± 0.1	4.1 ± 0.1
	Human	3.6 ± 1.2	3.6 ± 1.2	3.7 ± 1.03	3.8 ± 0.8	3.9 ± 0.9
	Animation	4.2 ± 0.2	4.3 ± 0.09	4.2 ± 0.03	4.3 ± 0.2	4.3 ± 0.2
A/G	Monkey	1.2 ± 0.1	1.2 ± 0.1	1.2 ± 0.1	1.2 ± 0.1	1.2 ± 0.1
	Human	1 ± 0.4	1 ± 0.5	0.9 ± 0.4	1 ± 0.4	0.9 ± 0.4
	Animation	1.2 ± 0.04	1.2 ± 0.06	1.2 ± 0.04	1.2 ± 0.06	1.2 ± 0.1
GLO	Monkey	3.5 ± 0.3	3.7 ± 0.4	3.6 ± 0.3	3.5 ± 0.2	3.5 ± 0.2
	Human	4 ± 0.7	4.1 ± 0.9	4.3 ± 1.01	4.3 ± 1.06	4.5 ± 0.1
	Animation	3.6 ± 0.2	3.6 ± 0.2	3.5 ± 0.2	3.6 ± 0.3	3.6 ± 0.3
AST	Monkey	43 ± 6.9	45.3 ± 11.4	42 ± 8.3	39.7 ± 7.8	39.3 ± 3.9
	Human	32.6 ± 6.03	32.7 ± 4.7	37.6 ± 6.4	35.7 ± 4.7	40.7 ± 13.2
	Animation	34.9 ± 4.5	34.1 ± 4.1	35.8 ± 7.4	34.1 ± 8.2	35.5 ± 9.4
ALT	Monkey	29.6 ± 4.7	31.7 ± 7.7	31.8 ± 6.2	31.8 ± 9.6	33.05 ± 9.5
	Human	33 ± 13.7	32.3 ± 12.4	34.9 ± 8.94	37.1 ± 7.4	39.7 ± 7.3
	Animation	34.3 ± 9.9	29.8 ± 6.3	30 ± 6.7	30.5 ± 8.2	34.8 ± 9.8
TBIL	Monkey	0.2 ± 0.005	0.2 ± 0.03	0.2 ± 0.03	0.2 ± 0.03	0.2 ± 0.01
	Human	0.1 ± 0.05	0.1 ± 0.06	0.2 ± 0.07	0.2 ± 0.03	0.2 ± 0.08
	Animation	0.2 ± 0.02	0.2 ± 0.03	0.2 ± 0.05	0.2 ± 0.05	0.2 ± 0.06
GGT	Monkey	77.2 ± 17.4	81.5 ± 13.6	82.4 ± 17.4	84.3 ± 18.3	81.2 ± 16.9
	Human	74.6 ± 44.6	77.9 ± 48.7	80.3 ± 43.6	81.6 ± 36.4	84.1 ± 36.7
	Animation	91.7 ± 17.5	95.6 ± 14.7	92.2 ± 10.1	101.2 ± 21.5	99.3 ± 15.8
ALP	Monkey	1364.6 ± 235.9	1332.3 ± 196.6	1335.9 ± 155.48	1344.4 ± 194.2	1338.2 ± 158
	Human	1473.9 ± 286.5	1433.4 ± 324.8	1372.2 ± 424.6	1377.2 ± 492.6	1486.5 ± 552.7
	Animation	1692.3 ± 407.7	1710 ± 399.8	1720.3 ± 451.4	1826.4 ± 406.4	1845.05 ± 361.4
CK	Monkey	160 ± 34.5	327.8 ± 318.1	172.5 ± 75	172 ± 45	158.3 ± 30.8
	Human	177.3 ± 63	154.3 ± 57.7	147.5 ± 32.2	153.8 ± 34.6	326.5 ± 272.6
	Animation	135.8 ± 11.8	201 ± 138.3	122.2 ± 37.8	128 ± 32.5	309 ± 336.1
LDH	Monkey	1106.5 ± 419	1061.5 ± 404.9	861.8 ± 232.1	743.8 ± 151.2	780.8 ± 144.1
	Human	923.5 ± 267.6	923.8 ± 432	1014.5 ± 360.1	825 ± 205.8	826.5 ± 342.3
	Animation	800 ± 194.8	785.3 ± 227	851.5 ± 191.8	730.3 ± 224.8	752.2 ± 210.6
TCHO	Monkey	149 ± 40.7	149.3 ± 30.9	153.3 ± 38.3	156.3 ± 47.4	152.5 ± 45.4
	Human	138 ± 14.3	136 ± 20.3	141 ± 22.7	144.8 ± 26.9	151 ± 24.7
	Animation	131.8 ± 48.7	138.3 ± 49.4	130.3 ± 46.3	143 ± 49.9	149.5 ± 51.3
TG	Monkey	30.8 ± 14.9	24.5 ± 9.9	24.3 ± 9.7	25.8 ± 9.7	15.7 ± 3.9
	Human	33.7 ± 25.8	45.6 ± 35.4	33.5 ± 17.8	32.5 ± 12.2	22.8 ± 11.1
	Animation	44.9 ± 24	28.3 ± 7.3	31.7 ± 9.3	27.1 ± 4.2	15.1 ± 1.1
Ca	Monkey	11 ± 0.5	10.8 ± 0.4	11 ± 0.1	10.9 ± 0.1	10.9 ± 0.3
	Human	10.3 ± 0.9	10.3 ± 0.8	10.3 ± 0.8	10.5 ± 0.7	10.6 ± 0.6
	Animation	10.6 ± 0.1	10.6 ± 0.3	10.4 ± 0.3	10.6 ± 0.4	10.7 ± 0.3
IP	Monkey	7 ± 1.9	7.6 ± 1.4	7.5 ± 1.3	7.4 ± 1.8	7.8 ± 1.1
	Human	7.01 ± 0.5	7.3 ± 0.5	7.1 ± 0.2	6.9 ± 1.2	7.7 ± 0.5
	Animation	6.4 ± 1.3	7.3 ± 0.3	7.2 ± 0.5	7.5 ± 0.6	7.5 ± 0.2
CRP	Monkey	0.2 ± 0.06	0.2 ± 0.02	0.2 ± 0.01	0.2 ± 0.03	0.2 ± 0.03
	Human	1.6 ± 2.8	0.6 ± 0.6	0.4 ± 0.5	0.2 ± 0.06	0.3 ± 0.2
	Animation	0.2 ± 0.02	0.2 ± 0.02	0.2 ± 0.01	0.2 ± 0.02	0.2 ± 0.01
Na	Monkey	148.3 ± 4.6	148 ± 3.2	149.5 ± 3.1	149.5 ± 2.4	149.8 ± 3.6
	Human	149 ± 3.4	149.5 ± 2.4	147.5 ± 3.1	149.5 ± 3.3	149 ± 2.6
	Animation	147.5 ± 0.6	148.5 ± 1.7	148.8 ± 0.5	148.8 ± 0.5	149.5 ± 1.3
K	Monkey	5.7 ± 0.6	5.5 ± 0.8	5.8 ± 1.4	5.3 ± 1.1	5.7 ± 0.8
	Human	5.5 ± 0.7	5.7 ± 0.8	5.4 ± 0.4	5.2 ± 0.4	5.7 ± 0.6
	Animation	5.5 ± 0.6	5.2 ± 0.2	5.3 ± 0.7	4.8 ± 0.5	5.6 ± 0.4
Cl	Monkey	103 ± 3.6	103.5 ± 3	104 ± 2.1	105 ± 1.9	106 ± 1.8
	Human	100 ± 3.6	107.8 ± 3	101.3 ± 2.9	102 ± 3.3	104.5 ± 1.9
	Animation	103 ± 1.8	103.5 ± 0.6	104.8 ± 1.3	104 ± 0.8	107.5 ± 1

Note: All are n.s. in statistical analysis.

### The mild increase in cortisol level is associated with motor planning-based cognitive behavior such as manipulation

**Figure 3:**
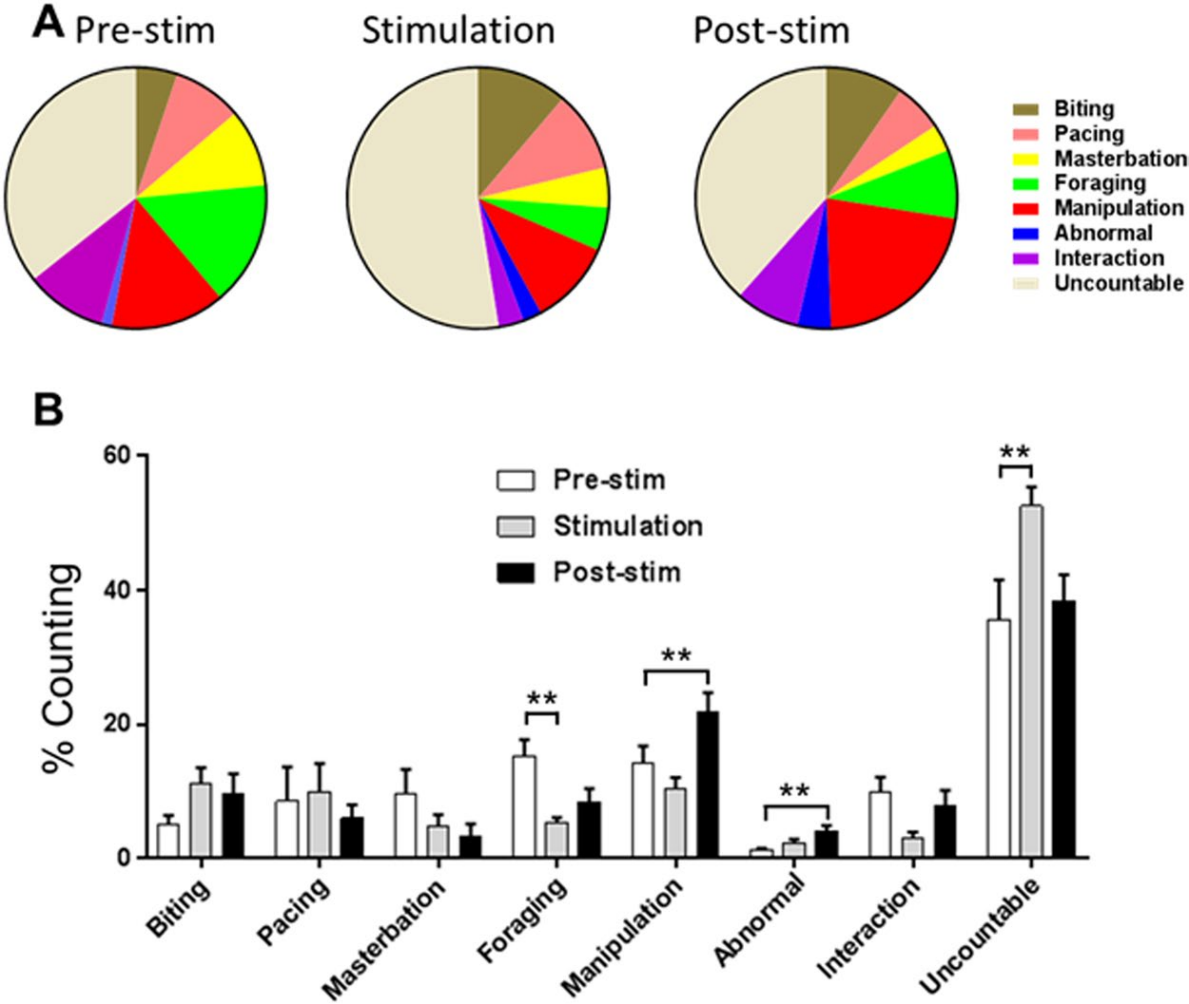
Behavioral changes. (**A**) Frequency of behavioral counting before, during, and after visual stimulation. Biting (brown), pacing (pink), masturbation (yellow), foraging (green), manipulation (blue), interaction with neighbor (purple), uncountable actions (gray) are pie chart for showing overall change in behaviors. (**B**) Foraging, behaviors using hand or mouth to put the food into mouth (one-way ANOVA F_2,35_ = 4.91, p = 0.020, followed by Dunnett’s post hoc test **p < 0.01). Manipulation, fingering, grasping of hands (one-way ANOVA F_2,35_ = 7.84, p = 0.0016, followed by Dunnett’s post hoc test **p < 0.01). Abnormal behavior, hit and suck by himself (one-way ANOVA F_2,35_ = 6.73, p = 0.0035, followed by Dunnett’s post hoc test **p < 0.01). Uncountable (one-way ANOVA F_2,35_ = 5.51, p = 0.0086, followed by Dunnett’s post hoc test **p < 0.01).

To assess the correlation between mild increase in cortisol level and behavioral changes, we performed in-depth off-line analysis of the video-recordings of individual monkey during each session. We classified each behavior as described in Table 3 based on behaviors as shown in Videos S1–S7, counted the frequency of each behavior and normalized to that of pre-stimulation week. And the percentage of each type of behavior was calculated from the total number of all behaviors. We found that Manipulation behavior was significantly increase during post-stimulation week compared to that of pre-stimulation week (Red pie, Fig. 3A). The behaviors that showed significant difference were Foraging, Manipulation, Abnormal, and Uncountable (Table 4). Only manipulation showed a significance difference at post-stimulation compared to pre-stimulation (One-way ANOVA F_2,26_ = 5.68, P = 0.0096, followed by Dunnett’s posthoc test, **p < 0.01, Fig. 3B and Table 4). The weight of monkey did not change significantly (Table 5), suggesting that mild increment of blood cortisol do not inhibit food uptake or metabolism, which are related to body weight. Consistent with increment of manipulation with the presence of visual stimulation, injections of cortisol and dexamethasone, Cortisol analogue, increased duration of manipulation behavior at 4 days-injection, not 11 and 18 days-injection (Fig. S1B and Videos S8 and S9). Manipulating behavior could be one of the strong candidates for measuring cognition, because objects can be differently handled by subject’s mental state^16^. Therefore the current results indicates that visual stimulation can change subject’s cognition including motor, emotion, and decision-making.

**Table 3:** Definition of behaviors.

Behaviors	Definition
Biting	Biting anything in cage
Pacing	Walking in the exact same pattern-either back and forth or in a circle 3 or more repetitions
Foraging	Picking up and placing a food item in the monkey's mouth (not including monkey chow in the cage)
Manipulating enrichment items	Any manipulation, movement, oral, or tactile exploration of puzzle ball, and supertube.
Abnormal behaviors	
- Self – suck	Sucking a part of the monkey's own body, including their digits, tail, or genitals for 3 or more seconds
- Self - hit	Any behavior involving forcibly hitting or slapping oneself on any part of the body
Interaction	Any behavior involving touching and grasping neighboring monkey
Masturbation	Self-directed stereotypies

**Table 4:** Summary for statistics of Fig. 3.

Behaviors	F	Significance
Manipulation	F_2,26_ = 5.68, P = 0.0096	**P < 0.01
Abnormality	F_2,26_ = 3.73, P = 0.039	**P < 0.01
Interaction	F_2,26_ = 2.53, P = 0.10	n.s.
Foraging	F_2,26_ = 7.46, P = 0.0030	**P < 0.01
Masturbation	F_2,26_ = 1.29, P = 0.29	n.s.
Pacing	F_2,26_ = 0.24, P = 0.78	n.s.
Biting	F_2,26_ = 1.76, P = 0.19	n.s.
Uncountable	F_2,26_ = 2.44, P = 0.11	**P < 0.01

**Table 5:** Individual weights were not changed along with the duration of the experiment.

Group (N) Weight (kg)	Pre-stim.	1^st^ stim.	2^nd^ stim.	3^rd^ stim.	Post-stim.
Monkey Pictures (4)	3.44 ± 0.21	3.42 ± 0.22	3.45 ± 0.23	3.48 ± 0.24	3.49 ± 0.20
Human Pictures (4)	3.43 ± 0.23	3.44 ± 0.30	3.44 ± 0.31	3.49 ± 0.29	3.50 ± 0.31
Animation Pictures (4)	3.43 ± 0.21	3.43 ± 0.25	3.46 ± 0.23	3.48 ± 0.26	3.49 ± 0.28

## Discussions

Here we propose periodical monitoring blood cortisol concentration to see whether single housed NHP is stressed or not, because the welfare of large laboratory animals is a very important factor when using them in preclinical settings. Increases in stress not only reduce the quality of welfare level, but also compromise the experimental results. This work used to measure blood cortisol concentration to monitor biological stress level which controlled by HPA axis. We found a mild increase in blood cortisol level ranging from 24–31% increases, suggesting that visual enrichment does not cause a severe stress (Fig. 2D). The previous reports have shown that cortisol level increases by 2 fold when monkeys are stressed by transportation^12^. Our results show that visual stimulation causes a mild stress as shown by a mild increase of about 24–31% in blood cortisol level and does not affect other stress indicator such as Creatine kinase by damage of skeletal muscle cells^20^.

Visual stimulation could be used as a means to environmental enrichment for C. monkeys housed in individual cage. Environmental enrichment is one of the important issues in the usage of captive non-human primates. Enriched environments provide opportunities for animals to engage more time in foraging, locomotor, and problem solving behaviors^21^. In other occasions, it has been shown that viewing consecutive videos increases play time duration and decreases abnormal behaviors^7^, and that removal of perches for gray-cheeked mangabey increased aggression^22^. Furthermore, environmental complexity such as adding woodchip, hiding foods, using frozen food improved food distribution and reduced fighting in non-human primates^23^. Visual stimulation has been proposed to be an effective way of enrichments for non-human primates. Socially housed bonnet macaques trained with joy stick to point out what they seek show the preference of conspecific video of new social group than own group, suggesting that subjects can differ visual stimulation and possess visual preference^24^. Visual stimulations composed of pictures showing threat, withdraw, fear, play, explore, and mother-infant showed innate behaviors in classification of isolating infant monkey, suggesting that certain communications lies in innate recognition mechanisms rather than in acquisition along with social learning processes from their family^3^. A previous study reported that a consecutive pictures combined with human, animation, and monkey had an effect on the changing a playing time and abnormal behaviors^7^. Therefore, our study suggests that visual stimulation can serve as one of the important factors for vision-mediated behavioral changes.

Cortisol has been associated with cognitive functions^13^. Our study is consistent with this idea and the mild level of blood cortisol is passively linked to cognitive behavior such as manipulation behavior. This mild level of cortisol appears to lead to more planning motor behaviors, which would be a physiological response relating to cognition for expecting next events which will happen shortly. Therefore, the slight increased level of blood cortisol would be a physiological response for cognitive enhancement. We expect that high blood cortisol level could potentially decrease cognitive behaviors. This exciting possibility should be tested in the future.

This study was based on the previous study by Ogura and Matsuzawa, who measured the frequency of abnormal behaviors by monitoring and counting monkey actions. They used visual stimulations composed of human picture, animation pictures, and monkey still pictures to see whether the frequency of the abnormal behavior reduced or not. Our study clearly demonstrated that visual stimulation improved the welfare of caged monkey by reducing the abnormal behaviors. We propose that mild stress by visual stimulation improves welfare of captive single-housed non-human primates.

## Materials and Methods

### Study site and Study subjects

The study was conducted on crab-eating monkeys (cynomolgus monkey, *Macaca fascicularis*) in Research Center for Animal Model, Korea Institute of Toxicology (KIT), which is located at 30 Baek Hak 1-gil, Jeongeup, Jeollabuk-Do, Republic of Korea. The crab-eating monkeys, which are laboratory non-primate from China (Guangxi Grandforest scientific primate company, Ltd., China), are housed in individual cages (510 W × 800 L × 764 H mm). Size of cage was satisfied the requirements for ‘The Guide for the Care and Use of Laboratory Animals (ILAR publication, 2010 National Academy Press. Twelve crab-eating monkeys participated in this experiment (Table 1). All were males. The room environment was automatically controlled 20~26 ℃, relative humidity 50 ± 10%, 12 hours light/12 hours dark cycle with 150~300 Lux, and ventilation 10~20 times/hour. Temperature and relative humidity was monitored and recorded daily. Animal room and cage cleaning was performed according to the Research Center for Animal Model’s standard operating procedure. The monkeys were provided food, Lab diet ® #5002, PMI Nutrition International, USA) at 9:00 a.m. and 6:00 p.m. and water ad libitum and were fed approximately 60 g of food (Certified Primate Diet #5048, PMI nutrition International, Inc.) twice a day. The animals were managed at KIT, an accredited animal facility, complying with the AAALAC International Animal Care Policies. The Animal Care and Use Committee of the KIT reviewed and approved all the study protocols.

### Videos for recording behaviors of C. Monkey

The apparatus in this study consisted of a color display TV (1101 × 661 × 95 mm, Samsung, model LN40T72BDA), which was connected to a laptop computer. The TV screen was located in the center of the cages of 4 individuals. The distance was 1.5 m. Subjects could not touch the TV or camera which was set up near the TV. The behaviors of each subject were recorded by a video camera (Sony HDR-CX130). During the experiment, all cameras ran for 3 hours. The group number and name of each subject was checked by performers and researchers when the experiments used to start.

### Apparatus for visual stimulation for C. Monkey

Each video was played in full screen mode, and 1 trial composed of 57 pictures. After one trial finished, there was a 3 seconds for black screen, in turn a one second beep sound. Then the next trial played. Fifty seven trial clips were shown in the first session. After first session finished, video clips for first trial were played again for second session. Thus, each subject was shown all of the video trials twice a day. Beginning of video clips was changed every 5 days because the novelty of the videos affects a macaque’s interest. Contents of video clips were classified as one of three types; monkey still pictures, human still pictures, and animation still pictures.

### Procedures

The experiment consisted of 3 observational phases conducted in the following order: pre-, during-, and post-stimulation. In the pre-stimulation phase, baseline data were collected without the visual stimulus and TV for four days, and then with a TV with blank screen for 4 days for habituating the monkey to the physical presence of the TV. After that, the stimulation phase was started in which video clips were played on the TV for 15 days. The ‘during stimulation” consisted of 3 types: consecutive human still pictures for Group 101, consecutive animation still pictures for Group 102 and consecutive monkey still pictures for Group 103. The videos were shown on the TV continuously for 2 hours a day. In the post-stimulation phase, a pre- stimulation period was conducted for 4 days. Two groups watched video clips from 9:00 am to 12:00 am and 1 group watched from 1:00 pm to 4:00 pm. Because cortisol concentration in blood is fluctuated^25^, times of collecting blood were at almost same time point. Experimental order was randomly mixed for reducing the variety of cortisol level. Video recording started immediately after subject was exposed visual stimulation. 3 hour-video recording per day composed of 2 hours for visual stimulation and 1 hour for blank TV.

### Hormone analysis

We obtained blood for analysis from samples that were collected for checking body condition. The analysis was conducted 5 times during study period from 12 March to 7 April. Hormone analyses were conducted at Sam-Kwang Medical Laboratories (Seoul, Korea). Before an assay, samples put into serum tube, were then mixed and centrifuged before 2 hours until analysis. We measured cortisol levels using Electro-chemiluminescence Immunoassay (ECLIA).

### Statistical analysis

One way ANOVA was used for multiple comparisons followed by Dunnette’s post-hoc test for each comparison in 3 groups (Figs 2 and 3). Paired student t-test was used for continuous blood cortisol concentration from same monkey along the interval described in Table 3. All data processing and statistical analysis were performed by using Prism 5 for Windows (Version 6.0 5 (trial), GraphPad Software Inc.).

## Electronic supplementary material

supplementary information

S1

S2

S3

S4

S5-1

S5-2

S6

S7

S8

S9
