# Supplementary material for: Visual stimulation-induced mild stress enhances cognitive behavior in cynomolgus monkey
Source: Sci Rep. 2018 Feb 28;8:3809. doi: 10.1038/s41598-018-22136-9 (PMC5830581; doi:10.1038/s41598-018-22136-9)
Supplement: Supplementary file 1 — supplementary information [file 41598_2018_22136_MOESM1_ESM.docx]

**Supplemental Information**

Title: Visual stimulation-induced mild stress enhances cognitive behavior in cynomolgus monkey

Authors: Dong Ho Woo, ^1^ Eun Ha Koh, ^4^ Seung-Hyuk Shin, ^3^ Young-Su Yang, ^1^ Jae Chun Choe, ^6^ C. Justin Lee, ^5^ Su-Cheol Han ^2,^*

Affiliations: ^1^Research Center for Animal Model, ^2^General Toxicology Research Center, ^3^Analytical Research Center, Jeonbuk Department of Inhalation Research, Korea Institute of Toxicology, KRICT, Jeongeup, Republic of Korea. ^4^Team of Research Planning and Management, Bureau of Ecological Research, National Institute of Ecology, Seocheon, Republic of Korea. ^5^Center for Glia-Neuron Interaction, Korea Institute of Science and Technology, Seoul, Republic of Korea. ^6^Laboratory of Behavior and Ecology, Division of EcoScience, Ewha Womans University, Seoul, Republic of Korea.

* Corresponding author

Su-Cheol Han, DVM, Ph.D.

General Toxicology Research Center

30 Baehak1-gil, Jeongeup, Jeollabuk-do,

53212, Republic of Korea

TEL: 82-63-570-8520

E-mail: vethansc@kitox.re.kr

**
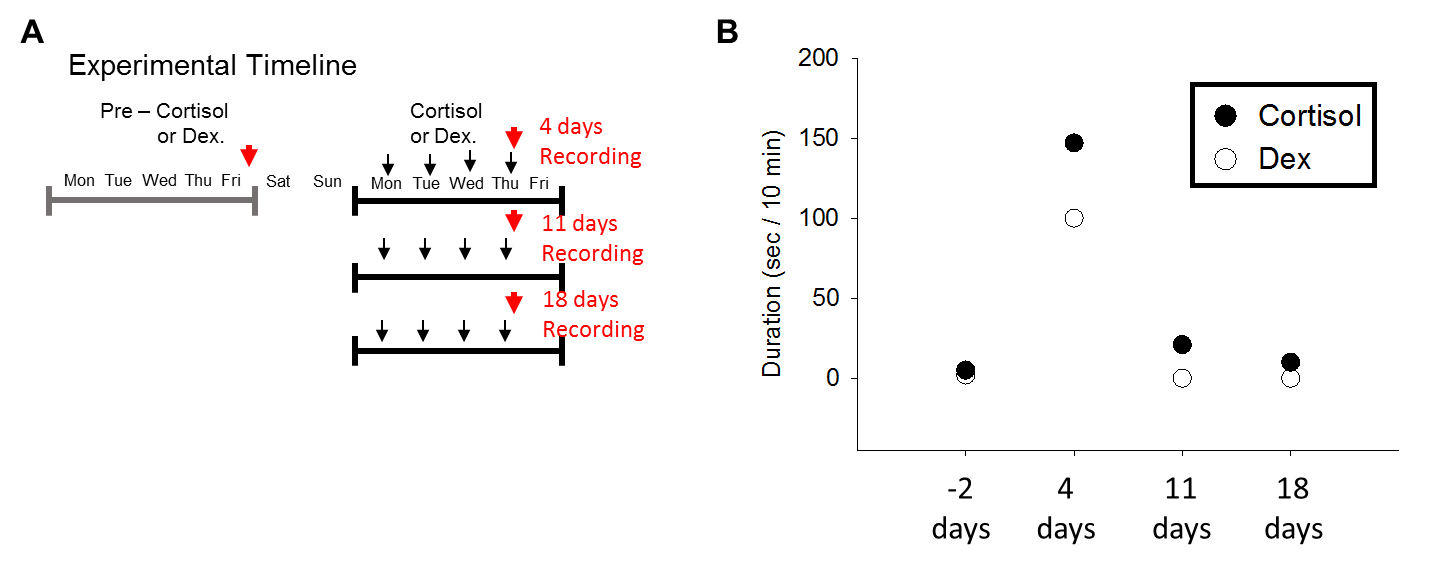
**

**Fig. S1. Cortisol and Dexamethasone (Dex), cortisol analogue, injections increase duration of manipulation behavior.** (A) Experimental timeline for the effect of cortisol injection on manipulation behavior. Red arrow for 10min video recording. Black arrow is for cortisol injection or Dex. (60 ng/ml, 4 times for 1 week) (B) Cortisol or Dex. injection increase manipulation behavior after 4 days injection but not 11 days and 18 days.

**Materials and methods for cortisol injection intravenously**

The KIT experimental number was B216070. To see whether that cortisol injections cause to increase the duration of manipulation behavior, one is for cortisol injection and the other was for Dex^1^. Injection. Sixty ng/ml Cortisol and Dex. were used because of around 60 ng/ml change of cortisol level from Figure 2D. A 10min video recording was for pre-injection and three 10min video recordings were for duration of injection. All basic results for the experiments were recorded on Pristima system (Version 7, Xybion Medical System Co., USA). For approving the publication of monkey image or recording file, we have approval number (No. 2018-01) from Image review committee of KIT. Eight animals were initially started but six animals were eliminated because they did not show any interesting for enrichment items.

**Video Legends**

1. Biting
2. Pacing
3. Foraging
4. Manipulation
5. Self-hit and suck
6. Self-biting

6. Interaction

7. Masturbation

**Reference**

1. Cole, M. A., Kim, J. K., Kalman, B. A., & Spencer, R. L. Dexamethasone suppression of corticosteroid secretion: evaluation of the site of action by receptor measures and functional studies. *Psychoneuroendocrinology* **25(2)**, 151-167 (2000).
